# A study analyzing macular microvasculature features after vitrectomy using OCT angiography in patients with idiopathic macular epiretinal membrane

**DOI:** 10.1186/s12886-020-01429-6

**Published:** 2020-04-22

**Authors:** Jianbo Mao, Jimeng Lao, Chenyi Liu, Caiyun Zhang, Yiqi Chen, Jiwei Tao, Lijun Shen

**Affiliations:** 1 Eye Hospital of Wenzhou medical university, Zhejiang, China; 2 Chicago College of Optometry, Midwestern University, Downers Grove, IL USA

**Keywords:** Macular microvasculature features, Retinal thickness, Idiopathic macular epiretinal membrane, Optical coherence tomography angiography, Vitrectomy

## Abstract

**Background:**

To evaluate postoperative changes in retinal capillary plexus and to assess contributing factors in postoperative visual improvement using optical coherence tomography angiography (OCT-A) in patients with idiopathic epiretinal membrane (iERM) post membrane removal.

**Methods:**

Patients scheduled for vitrectomy and membrane peel for iERM were enrolled. 35 subjects were included for this study. OCT-A was used to measure the FAZ related parameters and the superficial and deep capillary plexus layers using 3 mm × 3 mm scans. Measurements were taken before surgery and at every post-surgical follow-up. The unaffected fellow eyes were used as controls. Evaluated factors included: BCVA, vessel density (VD) and retinal thickness (RT) in five regions, FAZ area, FAZ perimeter (PERIM), acircularity index (AI) and foveal vessel density (FD).

**Results:**

Compared with the control group, the foveal vessel density (FVD) in superficial capillary plexus (SCP) was greater in the epi-retinal membrane group (*P* < 0.0001), whereas both groups had comparable parafoveal vessel density (PRVD) in SCP (*p* > 0.05). After surgery there was a reduction in the PRVD in SCP. The FVD in DCP increased and the PRVD in DCP decreased at baseline (*p* < 0.001). After surgery there was an increase in PRVD in DCP. By 6 months post-op, the PRVD had no statistically significant difference compared with the control group (*p* > 0.05). D-value of LogMAR BCVA was positively correlated with pre-op LogMAR BCVA (*p* < 0.0001), FVD in SCP (*p* < 0.001). It was negatively correlated with FAZ area (*P* < 0.001) and PERIM (*P* < 0.05).

**Conclusions:**

Vitrectomy and membrane removal led to the decrease of VD in SCP and the increase of PRVD in DCP. Patients with a more severe iERM may receive greater visual improvement with surgery.

**Trial registration:**

Trial registration number (TRN) and date of registration.

ChiCTR2000031289, retrospectively registered, 2020.03.26.

## Background

Idiopathic epiretinal membrane (iERM) is an avascular proliferative fibroblastic membrane located between the vitreous and the internal limiting membrane (ILM). It is a common cause of vision loss and metamorphopsia. Pars plana vitrectomy is the most effective method to improve visual function and quality of life [1, 2]. However, recovery after iERM surgery is a slow process [3, 4]. Optical coherence tomography (OCT) has been used to monitor the postoperative recovery, observing changes in central macular thickness, integrity of ellipsoid zone and other OCT based indicators. Damage in the parafoveal capillary plexus can be induced by the contraction of iERM, exerting anteroposterior and tangential forces on the retina. Fluorescein angiography (FA) can be used to detect fluorescein leakage in patients with iERM, along with vascular distortion, to evaluate disturbance in vascular hemodynamics [5, 6].

OCT angiography (OCT-A) represents a new imaging technique that allows for enface retinal vessel visualization without dye injection. It can be used to quantify the superficial and deep retinal capillary plexus (SCP and DCP) and evaluate the morphology of the fovea avascular zone (FAZ). Changes in microvasculature caused by iERM can be captured using OCTA. Nelis [7] and Kim [8] found that eyes with iERM can present with small FAZ area, increased foveal vessel density (FVD) and decreased parafoveal vessel density (PRVD), which reflecting the displacement of blood vessels into the fovea. Whether surgery can improve the displacement, there have been many studies [8–11]. Romano [9] found no significant differences in preoperative (pre-op) and postoperative (post-op) SCP, while Mastropasqua [10] observed a reduction in the vessel density (VD) in SCP at 1-month post-op. It was still controversial. Studies [8, 11] also found surgery resulted in a significant change both for FVD and PRVD at 6 months post-op, however the VD was still different from the fellow eyes.

Previous studies have well demonstrated some changes in macular microvasculature features in eyes with iERM between baseline and 6 months post-op. This study was designed in order to observe the longitudinal changes in macular microvasculature, central macular thickness and visual acuity after membrane peel at multiple post-ops. The consistency in capillary plexus changes was analyzed and OCT-A parameters as predictors for post-op visual acuity were investigated.

## Methods

Patients with iERM scheduled for vitrectomy were enrolled at the Eye Hospital of Wenzhou Medical University between March 2016 and December 2017. Patients were followed for 6 months after surgery. The study was adhered to the tenets of the Declaration of Helsinki. Regional Ethics Committee approval (2,019,168 K160) and parental consent were obtained in each case. Exclusion criteria were: (1) any previous ocular surgery in the study and fellow eyes; (2) any vitreoretinopathy secondary to causes other than iERM in the study and fellow eyes; (3) opacification of the refractive medium caused by any factors such as severe cataract.

All patients underwent 23-G pars plana vitrectomy. Internal limiting membrane was peeled with 0.25% indocyanine green dye staining in all cases. Phacoemulsification and foldable intraocular lens implantation were performed during vitrectomy. All operations were performed by the same chief surgeon with decades of surgical experience.

Best corrected visual acuity (BCVA) was measured at the baseline visit and every follow-up prior to slit lamp examination and dilated fundus exam with non-contact lens. OCT-A and fundus photography were also taken at the baseline visit and each follow-up. OCT-A (AngioVue, RTVue XR Avanti SD-OCT, Optovue, Fremont, CA, USA) was performed with 3 mm × 3 mm sections centered at the fovea. With three dimensional projection artefact removal (PAR) enabled software, projection artifact was removed from enface OCT-A images and B-scan images. OCT-A scans with a signal strength index lower than 6 out of 10, low quality images or unclear layer segmentation were excluded. The segmentations of SCP and DCP in each image were manually inspected and adjusted.

The main outcome measures were:

- VD and RT: both RT and VD of SCP and DCP were measured and analyzed in all five regions in the posterior pole (the temporal, superior, nasal, and inferior regions and the fovea area)
- FAZ parameters: the foveal capillary plexus discontinues at the margin of the macula, in a form of a ring, resulting in a capillary-free region called the fovea avascular zone
  - FAZ area or size
  - FAZ perimeter (PERIM)
  - Circularity index (AI): A-circularity index (AI) = measured perimeter / perimeter of regular circular with same FAZ area, reflecting the shape of FAZ
- FD: defined as the foveal vessel density in a 300 μm wide region around FAZ.
- Difference value (D-value) of LogMAR BCVA = (pre-op LogMAR BCVA) – (LogMAR BCVA at 6-month post-op)
- D-value of PRVD = (PRVD at 6-month post-op) – (pre-op PRVD)

The parameters were measured and analyzed in eyes with iERM as well as in the fellow eyes at 1-, 3-, 6-month(s) post-op. The last two parameters were collected at pre-op visit and 6-months post-op.

Statistical analysis was performed using the Statistical Package for Social Sciences (SPSS version 19.0, SPSS, Inc., Chicago, IL, USA). Data normally distributed were expressed as mean ± standard deviation (SD), if not, data were expressed as medians and interquartile range. One-way ANOVA test and non-parametric Kruskall-Wallis test were used to make comparisons among groups. The independent sample T test and Mann Whitney test were used for the comparison between the two groups. Spearman correlation analysis was used to analyze the correlation factors of D-value in LogMAR BCVA. *P* < 0.05 was considered to be statistically significant.

## Results

**Fig. 1:**
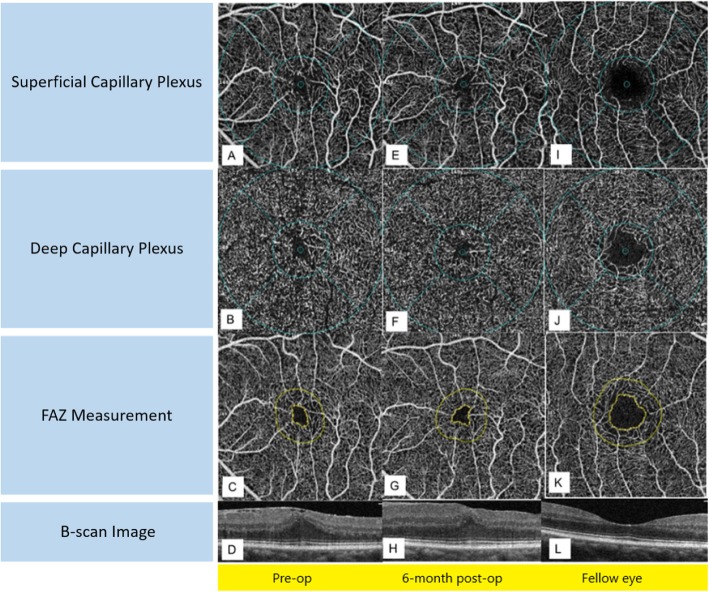
Pre- and post-op OCT-A images in a patient with iERM in the left eye

35 patients (9 men, 26 women) were included. The mean age was 64.18 ± 7.30 years (range from 47 to 78 years). In all, 35 eyes with iERM and 24 normal fellow eyes (control group) were collected. The LogMAR BCVA was 0.40 (0.30, 0.70) pre-op, 0.30 (0.22, 0.46) at 1-month, 0.22 (0.10, 0.30) at 3-month, and 0.22 (0.11, 0.30) at 6-month post-op. The LogMAR BCVAs at 3 months and 6 months were statistically different from pre-op baseline (*p* < 0.05). Figure 1 shows the OCT-A images of the iERM in a patient. The LogMAR BCVA was 0.22 pre-op and 0.10 at 6 months post-op. Both visual acuity and macular morphology improved.

### FAZ measurements

FAZ measurements were presented in Table 1. The mean FAZ area was 0.06(0.04,0.11) mm^2^ pre-op, which was smaller than (0.35 ± 0.13) mm^2^ (*p* < 0.0001) in the fellow eyes. Similarly, the FAZ PERIM pre-op was significantly smaller than the fellow eyes (*p* < 0.0001). During 6 months of follow-up, both the FAZ area and PERIM did not change significantly compared with pre-op measurements (*p* > 0.05). There was no statistical difference (*p* > 0.05) in AI between pre-op iERM (1.20 ± 0.07) and the fellow eyes (1.14 ± 0.03). After surgery, AI continuously increased by 6 months (1.22 ± 0.09), which was significantly higher than that in the fellow eyes (*p* < 0.05). No difference was found in FD between eyes with iERM and the fellow eyes (*p* > 0.05) or between pre- and post-op findings (*p* > 0.05).

**Table 1:** FAZ measurements

	FAZ area (mm^2^)	PERIM (mm)	AI	FD(%)
Control	0.35 ± 0.13	2.35 ± 0.48	1.14 ± 0.03	49.58 ± 3.38
Preop	0.06 (0.04,0.11)	1.11 ± 0.34	1.20 ± 0.07	48.15 ± 4.30
1 mo post-op	0.07 (0.04,0.09)	1.09 ± 0.34	1.18 ± 0.07	46.64 ± 5.20
3 mo post-op	0.07 (0.05,0.11)	1.16 ± 0.34	1.20 ± 0.07	48.31 ± 4.29
6 mo post-op	0.08 (0.04,0.13)	1.23 ± 0.41	1.22 ± 0.09	48.15 ± 4.13
F/K-W	35.10	33.86	3.39	1.13
P	< 0.0001	< 0.0001	< 0.05	0.35
Control vs. Pre-op	< 0.0001	< 0.0001	> 0.05	0.84
Pre-op vs. 6 mo post-op	0.36	0.83	> 0.05	> 0.99
Control vs. 6 mo post-op	< 0.0001	< 0.0001	< 0.05	0.87

*FAZ*: fovea avascular zone, *PERIM* FAZ perimeter, *AI* A-circularity index, *FD* foveal vessel density in a 300 μm wide region around FAZ, *Pre-op* preoperative, *1 mo post-op* 1 month postoperative, *3 mo post-op* 3 months postoperative, *6 mo post-op* 6 months postoperative

### Retinal thickness measurements

RTs in five regions were analyzed respectively. Measurements were presented in Table 2. RTs increased in all five regions in the iERM eyes compared to the fellow eyes (*p* < 0.0001). After pars plana vitrectomy, RTs in five regions all gradually decreased at each follow-up. At 6 months post-op, there was no statistical difference of the RTs in the temporal, superior and inferior regions compared to those at the control group (*p* > 0.05). The RTs in the fovea and the nasal region were significantly different from those in the control group (*p* < 0.05).

**Table 2:** Retinal thickness (RT) measurements

	Fovea RT (μm)	Tempo RT (μm)	Superior RT (μm)	Nasal RT (μm)	Inferior RT (μm)
Control	246.2 ± 22.7	319.5 ± 12.4	331.5 ± 13.2	333.1 ± 14.2	327.8 ± 15.4
Pre-op	446.4 ± 79.5	438.9 ± 65.8	437.2 ± 70.7	420.7 ± 64.2	405.9 ± 56.1
1 mo post-op	418.1 ± 47.0	359.7 ± 29.5	385.0 ± 32.4	399.7 ± 28.8	376.0 ± 28.7
3 mo post-op	404.5 ± 37.3	344.4 ± 27.3	369.4 ± 25.9	387.2 ± 24.9	358.0 ± 24.0
6 mo post-op	384.6 ± 40.3	326.9 ± 26.3	350.4 ± 25.8	372.1 ± 24.2	344.3 ± 24.4
F	37.8	34.5	20.47	13.73	15.51
P	< 0.0001	< 0.0001	< 0.0001	< 0.0001	< 0.0001
Control vs. pre-op	< 0.0001	< 0.0001	< 0.0001	< 0.0001	< 0.0001
Pre-op vs. 6 mo post-op	< 0.001	< 0.0001	< 0.0001	< 0.001	< 0.0001
Control vs. 6 mo post-op	< 0.0001	0.98	0.69	0.03	0.66

*Pre-op* preoperative, *1 mo post-op* 1 month postoperative, *3 mo post-op* 3 months postoperative, *6 mo post-op* 6 months postoperative

### Superficial capillary plexus measurements

**Fig. 2:**
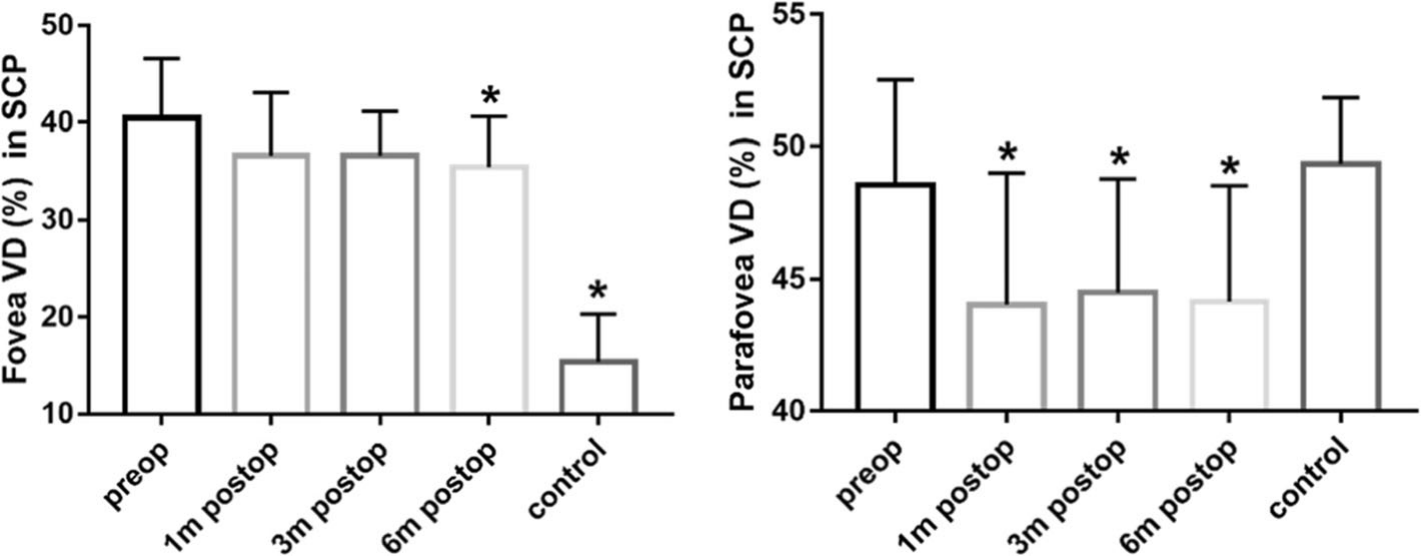
Foveal and parafoveal VD in SCP. Foveal (left) and parafoveal (right) vessel density (VD) in superficial capillary plexus (SCP) in eyes with iERM (preoperative, 1-month postoperative, 3-month postoperative, 6-month postoperative) and in the fellow eyes. * *p* < 0.05 vs the preoperative

Superficial capillary plexus measurements were presented in Table 3 and Fig. 2. Compared with the control group, the FVD increased (*P* < 0.0001), while the PRVD did not change significantly in iERM group (*p* > 0.05). Different from the other three regions, the mean VD in the nasal region pre-op had an increasing trend compared with the control group. After surgery there was a reduction in the VD in SCP, especially in the temporal region. At 6 months post-op, compared with the fellow eyes, PRVD in SCP decreased statistically (*p* < 0.05) besides the nasal region.

**Table 3:** Superficial capillary plexus (SCP) measurements

	Fovea VD (%)	Parafovea VD (%)	Tempo VD (%)	Superior VD (%)	Nasal VD (%)	Inferior VD (%)
Control	15.33 ± 5.01	49.36 ± 2.48	48.55 ± 3.05	50.85 ± 3.30	48.31 ± 2.54	49.74 ± 3.44
Pre-op	40.47 ± 6.13	48.54 ± 4.05	47.00 ± 4.43	49.49 ± 4.55	49.26 ± 5.18	48.42 ± 5.32
1 mo post-op	36.66 ± 6.42	44.03 ± 4.98	41.33 ± 5.37	45.28 ± 5.61	44.78 ± 5.04	44.28 ± 5.77
3 mo post-op	36.67 ± 4.52	45.69 ± 4.70	41.09 ± 4.71	45.87 ± 4.62	44.64 ± 5.28	45.82 ± 4.45
6 mo post-op	35.43 ± 5.23	44.14 ± 4.38	41.21 ± 5.07	45.24 ± 4.68	44.65 ± 5.22	44.00 ± 4.98
F	53.38	6.83	11.87	6.22	4.91	5.03
P	< 0.0001	< 0.0001	< 0.0001	< 0.001	< 0.01	0.001
Control vs. Pre-op	< 0.0001	0.97	0.84	0.90	0.97	0.93
Pre-op vs. 6 mo post-op	0.02	< 0.01	< 0.001	0.02	0.01	0.03
Control vs. 6 mo post-op	< 0.0001	< 0.01	0.0001	0.01	0.20	0.02

*Pre-op* preoperative, *1 mo post-op* 1 month postoperative, *3 mo post-op* 3 months postoperative, *6 mo post-op* 6 months postoperative

### Deep capillary plexus measurements

**Fig. 3:**
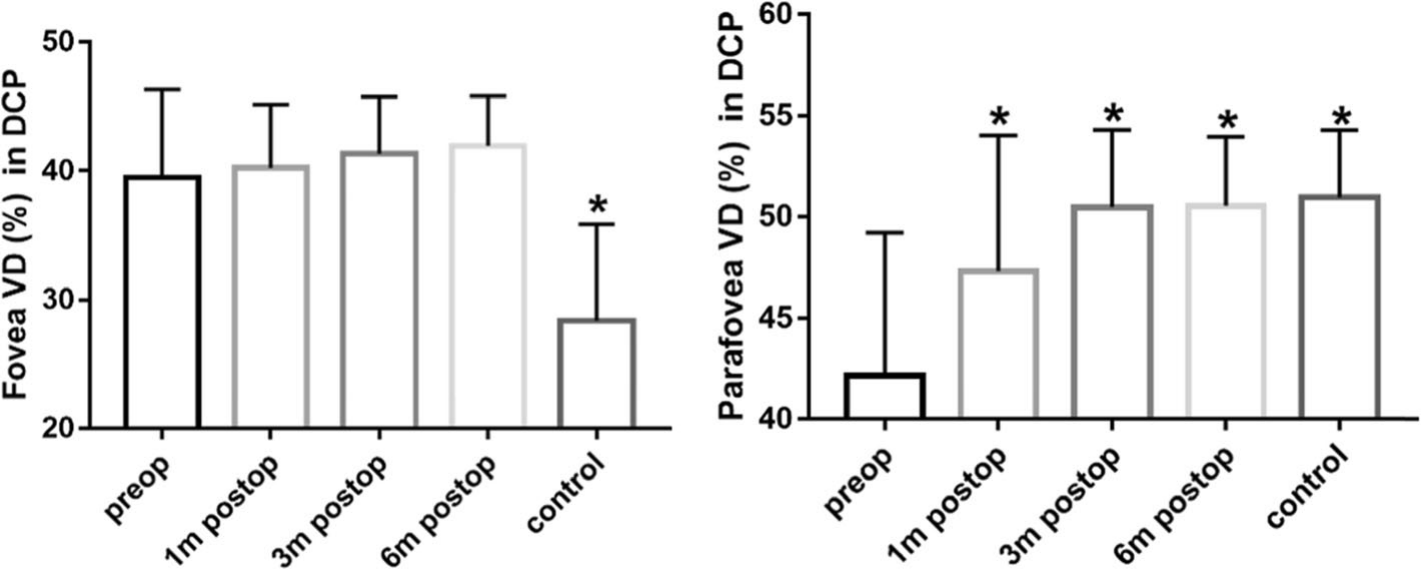
Foveal and parafoveal VD in DCP. Foveal (left) and parafoveal (right) vessel density (VD) in deep capillary plexus (DCP) in eyes with iERM (preoperative, 1-month postoperative, 3-month postoperative, 6-month postoperative) and in the fellow eyes. * *p* < 0.05 vs the preoperative

Deep capillary plexus measurements were presented in Table 4 and Fig. 3. The FVD increased, while the PRVD decreased (*p* < 0.001). The FVD had no statistically significant change (*p* > 0.05) at each post-op. Meanwhile, the PRVD in all regions slowly increased after the operation (*p* < 0.01). At 6-month post-op, the PRVD had no statistically significant difference compared with those in the control group (*p* > 0.05), which suggested that the PRVD had recovered to normal after surgery.

**Table 4:** Deep capillary plexus (DCP) measurements

	Fovea VD (%)	Parafovea VD (%)	Tempo VD (%)	Superior VD (%)	Nasal VD (%)	Inferior VD (%)
Control	28.39 ± 7.46	50.96 ± 3.33	51.97 ± 3.27	50.62 ± 4.12	51.25 ± 3.48	50.00 ± 3.63
Pre-op	39.49 ± 6.83	42.66 ± 6.63	42.83 ± 7.13	40.87 ± 9.58	43.27 ± 7.89	42.11 ± 6.79
1 mo post-op	40.25 ± 4.91	47.29 ± 6.73	48.63 ± 5.99	46.50 ± 7.28	47.14 ± 7.07	46.12 ± 7.58
3 mo post-op	41.35 ± 4.43	50.47 ± 3.84	50.79 ± 3.47	50.51 ± 4.01	49.58 ± 4.13	50.90 ± 4.44
6 mo post-op	41.96 ± 3.86	50.54 ± 3.43	51.61 ± 3.29	50.65 ± 3.79	49.41 ± 3.64	50.06 ± 4.11
F	15.96	10.21	12.87	10.21	6.38	9.36
P	< 0.0001	< 0.0001	< 0.0001	< 0.0001	0.0001	< 0.001
Control vs. pre-op	< 0.0001	< 0.0001	< 0.0001	0.0001	< 0.001	0.0001
Pre-op vs. 6 mo post-op	0.55	< 0.0001	< 0.0001	< 0.0001	< 0.01	< 0.0001
Control vs. 6 mo post-op	< 0.0001	> 0.99	> 0.99	> 0.99	0.89	> 0.99

*Pre-op* preoperative, *1 mo post-op* 1 month postoperative, *3 mo post-op* 3 months postoperative, *6 mo post-op* 6 months postoperative

### Correlation analysis of D-value of LogMAR BCVA

D-value of LogMAR BCVA can indicate the degree of visual improvement after surgery. It was positively correlated with preoperative LogMAR BCVA (*p* < 0.0001) and FVD in SCP (*p* < 0.001). It was negatively correlated with FAZ area (*P* < 0.001) and PERIM (*P* < 0.05) (Table 5). Thus, eyes with smaller FAZ area, smaller PERIM and worse vision will have greater visual improvement.

**Table 5:** Correlation analysis of D-value of LogMAR BCVA

	rho	*p*	rho	*p*
	Baseline		6 months post-op	
LogMAR BCVA	0.692	< 0.0001*	−0.256	0.138
FAZ measurements				
area	−0.536	< 0.001*	− 0.283	0.100
PERIM	−0.505	< 0.05*	− 0.214	0.216
AI	0.179	0.304	−0.017	0.923
FD	0.264	0.125	0.084	0.631
SCP VD				
Fovea	0.584	< 0.001*	0.200	0.250
Parafovea	0.024	0.890	0.212	0.222
DCP VD				
Fovea	0.129	0.460	0.310	0.070
Parafovea	−0.173	0.321	0.238	0.168
D-value of Parafovea VD				
SCP	0.222	0.199		
DCP	0.243	0.159		

D-value of LogMAR BCVA = preoperative LogMAR BCVA - LogMAR BCVA at 6-month post-op; D-value of PRVD = PRVD at 6-month post-op - preoperative PRVD

## Discussion

This study presented the sequential changes in macula microvasculature after vitrectomy with membrane peeling and analyzed the correlating factors in postoperative visual improvement.

In this study, the pre-op FAZ area and PERIM in iERM eyes were smaller than those in the fellow eyes. Romano [9] found no significant difference in FAZ area in iERM between pre-op and 6-month post-op. Kitagawa [12] found mean superficial FAZ area and deep FAZ area both increased (to 0.080 mm^2^ and 0.113 mm^2^ respectively) at 6 months after vitrectomy. However, the expansion was relatively small compared with the fellow eyes. Kumagai [13] studied superficial FAZ after surgery and found that the post-op FAZ areas in both iERM and macular hole groups were significantly smaller than that in the healthy control group (0.148 ± 0.094 and 0.255 ± 0.111 vs. 0.358 ± 0.118 mm^2^). The above results showed that surgery resulted in limited improvement in the centripetal vascular displacement caused by ERM, which was consistent with our study. In addition, AI was significantly greater after surgery, which indicated that the centripetal contractile force was inconsistent in different directions after internal limiting membrane peeling.

RT was observed pre- and post-op. After pars plana vitrectomy, all RTs decreased gradually. ERM formation exposes the retina to anteroposterior force, which leads to the thickening of macula. Many studies [14, 15] have covered the changes in fovea thickness assessed by OCT imaging. In this study, what’s special was that RT was separately calculated in five regions. At the end, we found that the foveal and nasal RT had minimal change, but the rest returned to normal thickness. While it is difficult to gain full surgical restoration, the regional difference in RT recovery may lie in its adjacent structure, such as the optic nerve to take example of the nasal region.

We were able to demonstrate the increase in FVD in SCP, which is in correlation to the smaller FAZ area as a result of the contraction from the periphery to the center of the membrane. At 6 months post-op, the VD in SCP decreased statistically. This result indicated that the membrane removal resulted in decreased VD in SCP. First, the iERM is located at the interface between the inner retina and the vitreous causing greater damage to the SCP, which is hard to recover even after surgery. Secondly, the strong adhesion between Müller cells and the ILM may result in ultrastructural damage to the inner retinal surface during the ILM peeling [16]. Mastropasqua [10] pointed out that the reduction of VD in SCP after surgery could correspond to the swelling of the arcuate nerve fiber layer due to the direct surgical trauma to the inner retina during the ILM grasping. However, the study of Chen [11] showed increase in the parafoveal VD in SCP after vitrectomy. Internal limiting membrane was peeled with 0.25% indocyanine green dye staining in all cases in our study, which may aggravate the damage to the SCP.

Changes in both the SCP and RT in the nasal region were distinctive, which could be related to its particular position or the direction the surgeon used to peel off the membrane. This specific principle needs further study.

As for DCP, there has been little research in the past. We found the increase in FVD, while the PRVD decreased in iERM group. At 6 months post-op, the PRVD in DCP increased significantly. In Lin’s study [17], the hypofluorescent areas on the fluorescein angiography corresponded to areas with reduced flow signal in the DCP on OCT-A scans in patients with iERM, which led to the proposal that the DCP can be damaged by the mechanical traction from iERM but can also be surgically restored. This potentially explained the pre-op low PRVD and the post-op improved or increased PRVD in DCP. In addition, unlike SCP, there was no direct iatrogenic injury during the ILM grasping.

In the past, a large number of studies [18–21] have focused on the changes in microstructure of fovea due to iERM in order to identify the anatomic changes associated with the vision recovery after surgery. This included the integrity of ellipsoid zone, the central macular thickness (CMT) and the photoreceptor outer segments. Few studies evaluated changes using OCT-A. In this study, positive correlations were identified between smaller FAZ area, smaller PERIM and greater visual improvement. In theory, a more severe iERM could ultimately cause diminution of the FAZ area due to the contraction and centripetal force. The results above may suggest greater visual outcome or higher proportion of visual improvement with more severe iERM, which places a favor in later surgical intervention.

## Conclusions

OCT-A is a useful tool to monitor post-op changes in the iERM and potentially predict visual recovery. At 6 months post-op, the RT returned to normal besides the nasal and foveal regions. The membrane removal led to the decrease of VD in SCP and the increase of PRVD in DCP. Patients with a more severe iERM may receive greater visual with surgery. However, there were several limitations to our study. Firstly, this paper only used the BCVA as the observation index for visual improvement. In the future, we hope to include the change in microperimetry and metamorphopsia. Several traditional OCT factors, such as ellipsoid zone and cone outer segment tip length, were also not discussed in our study given its focus on use of OCT-A, which can be included in our future study. Secondly, this article only studied patients with internal limiting membrane peeling. In future, we hope to observe changes in iERM using OCT-A after pars plana vitrectomy without internal limiting membrane peeling.

## Data Availability

The datasets used and/or analyzed during the current study are available from the corresponding author on reasonable request.

## Abbreviations

iERM: Idiopathic epiretinal membrane
ILM: Internal limiting membrane
OCT: Optical coherence tomography
FA: Fluorescein angiography
OCT-A: OCT angiography
SCP and DCP: Superficial and deep retinal capillary plexus
FAZ: Fovea avascular zone
VD: Vessel density
FVD: Foveal vessel density
PRVD: Parafoveal vessel density
pre-op: Preoperative
post-op: Postoperative
BCVA: Best corrected visual acuity
RT: Retinal thickness
PERIM: FAZ peripmeter
AI: Acircularity index
D-value: Difference value
